# MicroRNA-Mediated Regulation of Ionizing Radiation Responses: Mechanisms and Advances in Clinical Translation

**DOI:** 10.3390/cimb48090864

**Published:** 2026-08-25

**Authors:** Keying Lian, Quan Ma, Xiao Mo, Zhisheng Jiang, Yun Ma

**Affiliations:** 1 Institute of Biochemistry and Molecular Biology, Hengyang Medical College, University of South China, Hengyang 421001, China; lianky0522@163.com (K.L.); mx231275639@outlook.com (X.M.); jzsnhdx@126.com (Z.J.); 2 Nuclear Power Institute of China, Chengdu 610213, China; mq94080@163.com; 3 Department of Biochemistry and Molecular Biology, Laboratory of Nuclear Radiation DNA Damage and Repair, School of Basic Medicine, Hengyang Medical School, University of South China, Hengyang 421001, China

**Keywords:** microRNA, ionizing radiation, DNA damage response, radiosensitivity

## Abstract

Ionizing radiation can induce DNA damage through direct or indirect mechanisms and activate the DNA damage response network, thereby influencing cellular repair, cell cycle regulation and cell fate determination. MicroRNAs (miRNAs), as key post-transcriptional regulatory factors, play a vital role in modulating the radiation response and cell fate. This review summarizes radiation-induced changes in miRNA expression and the molecular regulatory mechanisms they mediate, and further discusses recent advances in their application in the assessment of radiation damage, the prediction of radiotherapy response, and therapeutic interventions. Although miRNAs possess significant translational value, their multi-target regulatory characteristics, tissue- and environment-dependence, as well as limitations in clinical detection and delivery systems, continue to hinder their application. In the future, the integration of multi-omics technologies, artificial intelligence analysis and precision delivery strategies is expected to further elucidate the miRNA-mediated radiation response network and advance their application in precision radiology.

## 1. Introduction

**Figure 1:**
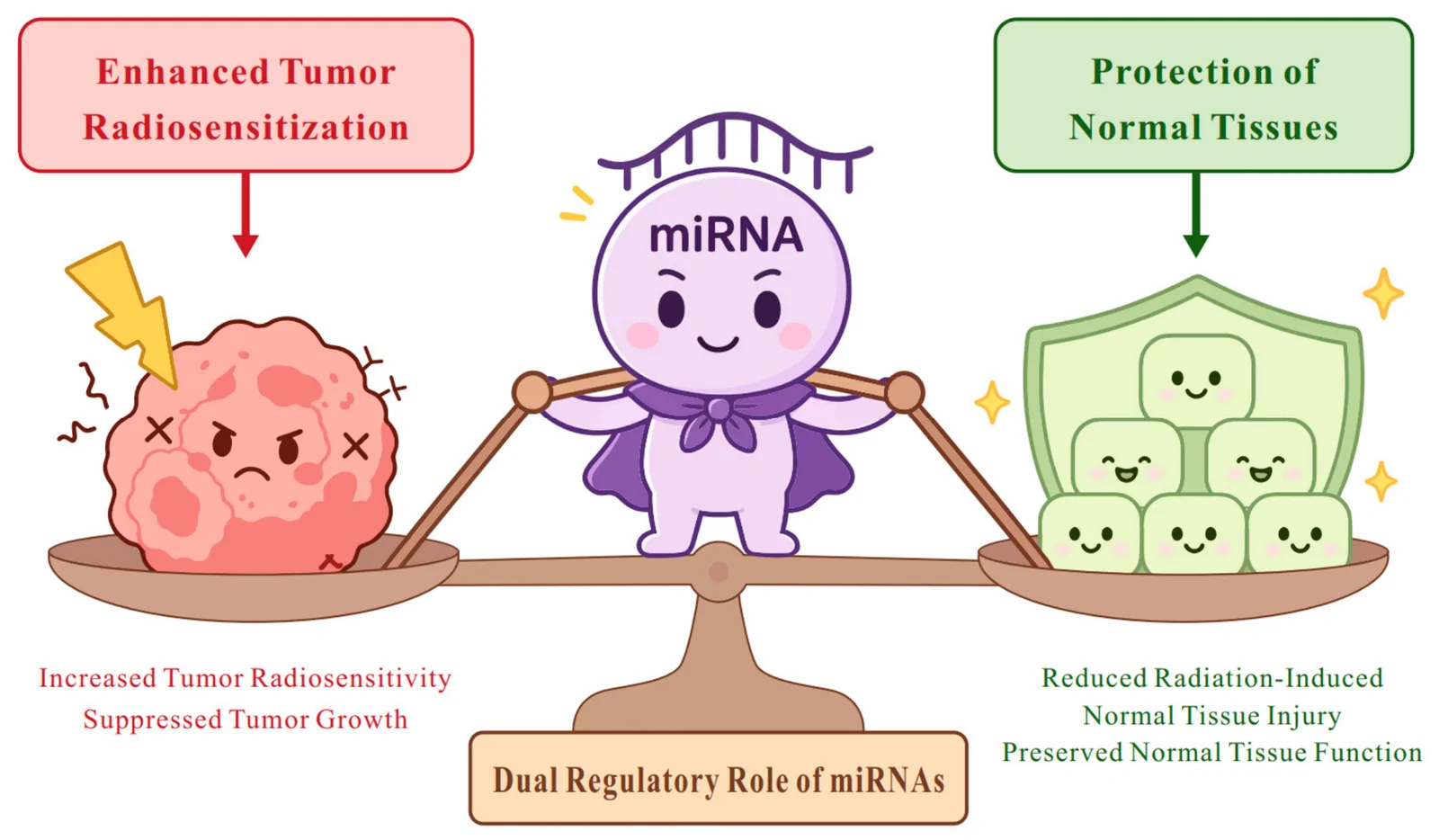
miRNAs play a dual role, both promoting damage and providing protection.

Ionizing radiation is widely used in radiotherapy for cancer and in medical diagnostics; however, the DNA damage it induces remains a significant factor affecting treatment efficacy and causing damage to normal tissues. Ionizing radiation can induce DNA damage and activate the DNA damage response (DDR) network either by acting directly on DNA or by inducing the production of reactive oxygen species (ROS). With the advancement of single-cell sequencing and multi-omics technologies, radiation biology has entered a phase of refined molecular analysis, revealing that the radiation response exhibits cellular heterogeneity and complex regulatory characteristics. However, the identification of key regulatory nodes and their clinical application remain significant challenges in current research. MicroRNAs (miRNAs) are a class of endogenous non-coding RNAs approximately 18–25 nt in length. As important post-transcriptional regulatory molecules, they possess multi-target regulatory capabilities and can participate in DNA repair, oxidative stress, cell cycle and cell fate regulation by modulating DDR-related genes. Notably, miRNAs exhibit a dual role in the radiation response: some miRNAs enhance tumor radiosensitivity, whilst others contribute to the radiation protection of normal tissues (Figure 1) [1,2]. Although previous studies have revealed the important role of miRNAs in the radiation response, there remains a lack of systematic summaries regarding their regulatory networks and clinical application value. Therefore, this review summarizes the molecular mechanisms by which miRNAs regulate radiosensitivity, outlines their applications in radiological medicine, and analyses current limitations and future directions.

## 2. Radiation-Induced Re-Profiling of miRNA Expression

Ionizing radiation can cause a re-profiling of the intracellular miRNA expression profile by influencing the transcriptional regulation, processing, and stability of miRNAs. This change is not merely a concomitant phenomenon of radiation damage but is influenced by a combination of factors, including the DDR, oxidative stress, and epigenetic regulation, resulting in radiation response patterns with specific biological significance. This response pattern exhibits distinct dose-, time-, and cell-type-dependent characteristics; different irradiation doses and tissue origins can lead to distinct miRNA expression profiles. Whilst some miRNAs respond rapidly in the early stages of radiation exposure and participate in the regulation of acute stress, others undergo sustained changes that may further influence the maintenance of cellular homeostasis, damage repair, and radiosensitivity [3,4,5]. Consequently, the dynamic expression patterns of miRNAs not only reflect the process by which cells adapt to radiation stimulation but may also serve as an important molecular basis for regulating cell fate. Furthermore, radiation-induced miRNA changes possess dual attributes as both intracellular regulators and extracellular biomarkers. Intracellularly differentially expressed miRNAs may participate in the regulation of radiation response networks, whilst miRNAs released into the circulatory system can stably carry molecular information related to radiation damage, demonstrating potential value for liquid biopsy applications in the assessment of radiation exposure, prediction of tissue damage, and monitoring of radiotherapy response [3,4].

## 3. Mechanisms Underlying miRNA-Mediated Radiosensitisation

The functions of miRNAs in the cellular response to ionizing radiation extend far beyond individual signaling pathways, encompassing DNA damage repair, cell fate determination, and intercellular communication. Through multilayered regulatory networks, miRNAs integrate radiation-induced stress signals and coordinate adaptive responses that ultimately determine cellular radiosensitivity and the severity of radiation-induced tissue injury [2,6,7].

### 3.1. Cell Cycle Regulation and DNA Damage Repair

**Figure 2:**
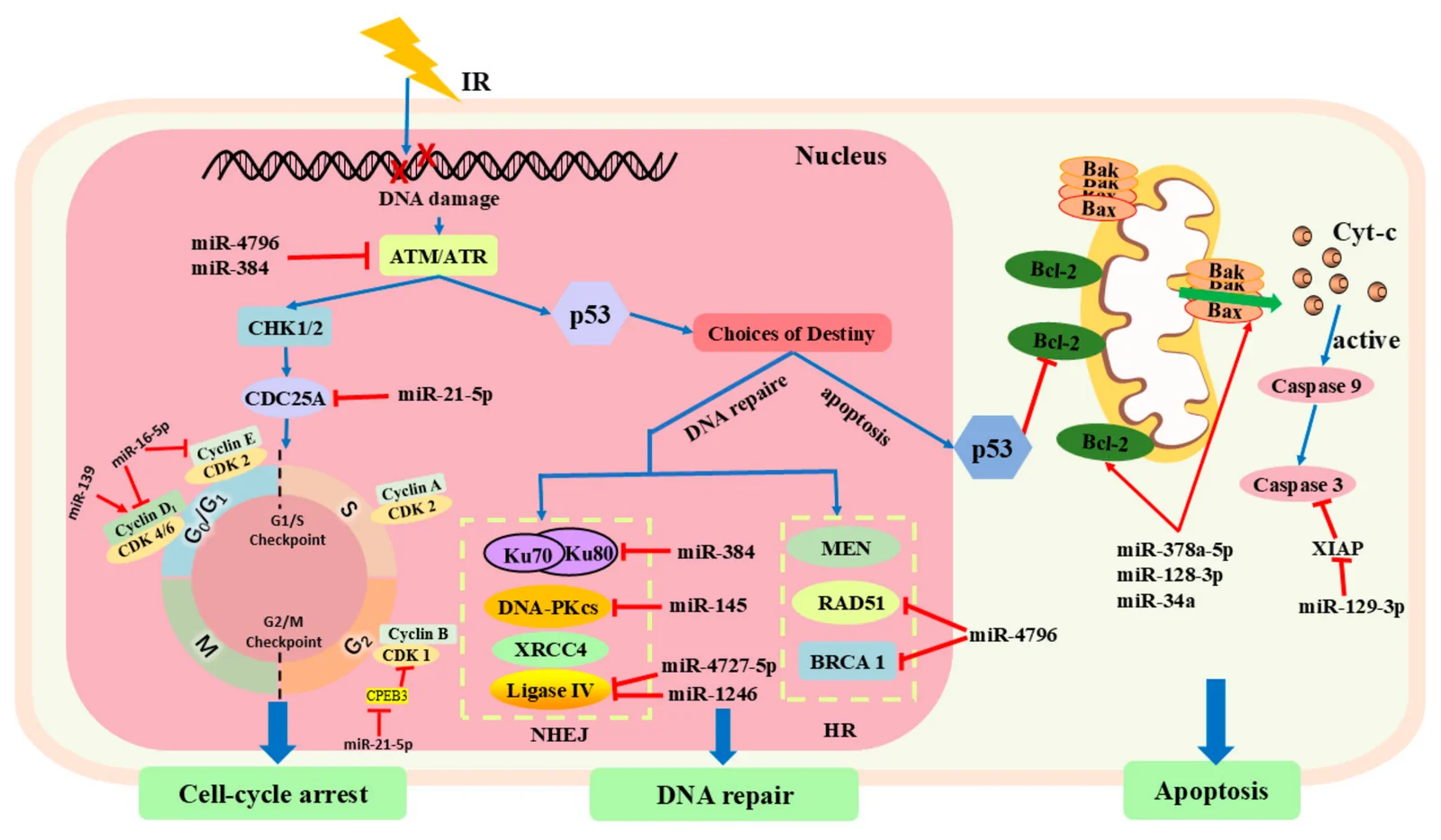
miRNA-mediated regulation of radiation-induced cellular responses. Ionizing radiation induces DNA damage and activates DDR signaling. Radiation-responsive miRNAs regulate DNA damage sensing, repair pathway selection between HR and NHEJ, and downstream cell fate decisions, including apoptosis, autophagy, and ferroptosis. Through these mechanisms, miRNAs determine tumor cell survival and modulate radiosensitivity.

The DDR constitutes the principal surveillance system responsible for detecting DNA lesions, coordinating cell cycle progression, and facilitating DNA repair. As key post-transcriptional regulators, miRNAs fine-tune multiple stages of the DDR, including damage sensing, checkpoint activation, and DNA repair pathway selection, thereby shaping cellular responses to ionizing radiation. At the initiation of the DDR, miRNAs regulate the magnitude and duration of DNA damage signaling by targeting core sensors and signaling kinases. For example, miR-384 directly suppresses the expression of ATM, thereby attenuating DNA repair capacity and increasing cellular radiosensitivity [8]. In addition, several miRNAs directly or indirectly regulate the MRE11–RAD50–NBS1 (MRN) complex, the primary sensor of DNA double-strand breaks (DSBs), thereby influencing downstream ATM-dependent signaling and subsequent repair responses. Following DDR activation, transient cell cycle arrest at the G1/S or G2/M checkpoint provides cells with an opportunity to repair damaged DNA before DNA replication or mitotic entry. miRNAs contribute substantially to checkpoint regulation by targeting cyclins, cyclin-dependent kinases, and checkpoint-associated signaling molecules. At the G1/S transition, miR-16-5p promotes G0/G1 arrest through inhibition of Cyclin D1, Cyclin E1, and the Rb–E2F1 signaling axis, thereby limiting the replication of damaged DNA [9]. miR-21-5p can influence G2/M phase arrest by modulating the CPEB3/CDK1/Cyclin B axis, maintaining cell cycle arrest following DNA damage [10]. In contrast, certain miRNAs facilitate checkpoint bypass. Under irradiation, miR-101-3p suppresses Wee1, abbreviates G2/M arrest, and drives hepatocellular carcinoma cells into premature mitosis, ultimately enhancing radiation-induced cell death [11]. Repair pathway selection following DSB formation represents another critical determinant of radiation sensitivity. Cells primarily repair DSBs through homologous recombination (HR) or non-homologous end joining (NHEJ), and accumulating evidence indicates that miRNAs are important regulators of this pathway choice. Several miRNAs, including miR-384 and miR-4796, target key HR factors such as BRCA1, BRCA2, and RAD51, thereby suppressing HR efficiency and promoting a greater dependence on NHEJ or resulting in defective DNA repair [8,12]. Conversely, other miRNAs preferentially impair NHEJ by targeting its core components. For instance, miR-145 directly inhibits DNA-PKcs [13], whereas miR-4727-5p and miR-1246 suppress DNA ligase IV [14,15], leading to compromised NHEJ activity, persistent DSB accumulation, and increased radiosensitivity. Importantly, miRNAs dynamically coordinate the balance between HR and NHEJ by regulating the expression of multiple repair proteins, thereby influencing repair fidelity, genomic stability, and cell survival after irradiation. In oral squamous cell carcinoma, miR-21-5p enhances PARP1- and POLQ-dependent signaling, promoting reliance on the alternative end-joining pathway and consequently contributing to radioresistance [16]. Collectively, these findings demonstrate that radiation-responsive miRNAs function as central modulators of the DDR by integrating DNA damage signaling, checkpoint control, and repair pathway selection, thereby exerting profound effects on cellular radiosensitivity (Figure 2).

### 3.2. Cell Fate Determination

When radiation-induced DNA damage exceeds the cellular repair capacity, cells initiate fate-determination programs that dictate whether damaged cells survive or undergo programmed elimination. As a pivotal event following DNA damage repair, cell fate determination reflects a dynamic balance between survival-promoting and death-inducing signaling networks. By coordinately regulating apoptosis, autophagy, and ferroptosis, miRNAs function as key post-transcriptional modulators that fine-tune these opposing processes and ultimately influence cellular radiosensitivity (Table 1). Apoptosis represents the primary mechanism for eliminating irreparably damaged cells following irradiation. Radiation-responsive miRNAs regulate both intrinsic and extrinsic apoptotic pathways through modulation of p53, Bcl-2 family proteins, and the caspase cascade. Depending on their molecular targets, certain miRNAs promote apoptosis by enhancing pro-apoptotic signaling, thereby increasing radiosensitivity, whereas others suppress apoptotic signaling to facilitate cell survival and contribute to radioresistance [17,18,19,20,21]. The balance between these opposing regulatory effects plays a critical role in determining cellular responses to radiation-induced stress. Autophagy constitutes another important adaptive response to radiation stress and exhibits context-dependent functions in determining cell fate. Basal or moderate autophagy promotes the degradation of damaged organelles and protein aggregates, thereby maintaining cellular homeostasis and facilitating survival under stress conditions. In contrast, excessive or persistent autophagic activity may contribute to autophagy-dependent cell death. Radiation-responsive miRNAs regulate autophagic flux by targeting key components of the autophagy machinery, including Beclin-1, ATG family proteins, and the mTOR signaling pathway, thereby modulating cellular tolerance to radiation-induced injury [22,23,24,25]. More recently, ferroptosis has emerged as an important mechanism underlying radiation-induced cell death. Unlike apoptosis or autophagy, ferroptosis is characterized by iron-dependent lipid peroxidation resulting from disrupted redox homeostasis. Because ionizing radiation generates excessive reactive oxygen species and promotes oxidative stress, it provides a favorable biochemical environment for ferroptosis. Increasing evidence indicates that miRNAs further regulate this process by targeting critical ferroptosis-associated molecules. For example, miR-320a indirectly suppresses GPX4 through regulation of RAD51, thereby promoting lipid peroxidation and ferroptotic cell death. Likewise, miR-214 directly targets GPX4 while also regulating SLC7A11, collectively enhancing the radiosensitivity of tumor cells [26]. Taken together, radiation-responsive miRNAs orchestrate multiple cell fate programs by integrating apoptotic, autophagic, and ferroptotic signaling pathways. Through coordinated regulation of these interconnected processes, miRNAs determine the balance between cellular survival and programmed cell death following irradiation, thereby exerting profound effects on radiosensitivity and the biological consequences of radiation exposure.

### 3.3. Interplay Among Non-Coding RNAs and Extracellular Vesicle-Mediated Propagation of Radiation Responses

Radiation-induced damage is not restricted to directly irradiated cells but can also propagate to neighboring non-irradiated cells through the radiation-induced bystander effect (RIBE). Increasing evidence indicates that non-coding RNA networks contribute to this non-cell-autonomous response by regulating intracellular signaling and facilitating intercellular communication. In particular, miRNAs participate in competitive endogenous RNA (ceRNA) networks within cells and are transported through extracellular vesicles (EVs), thereby expanding the regulatory scope of radiation responses (Table 2). Ionizing radiation reshapes the expression landscape of lncRNAs and circRNAs, which subsequently modulate miRNA activity through ceRNA mechanisms. Radiation-responsive lncRNAs, including NORAD, TRERNA1, and HOTAIR, can regulate DNA repair, autophagy, and cell survival by competing for miRNA binding and altering miRNA availability [35,36,37,38,39,40]. Similarly, radiation-associated circRNAs influence epithelial–mesenchymal transition, migration, and invasion through miRNA-mediated regulatory networks, thereby contributing to malignant progression after radiotherapy [41,42,43,44,45,46]. Unlike direct miRNA–target interactions, ceRNA regulation depends on the overall composition and dynamic balance of competing transcripts. Therefore, the biological outcome of a given miRNA is determined not only by its expression level but also by the regulatory state of the surrounding RNA network.

In addition to intracellular ceRNA regulation, EV-mediated transfer provides an important mechanism for long-range propagation of miRNA-mediated radiation responses. Irradiated cells selectively package specific miRNAs into EVs and deliver them to recipient cells, where these transferred miRNAs regulate DNA damage repair, cell cycle progression, autophagy, ferroptosis, and other stress-response pathways. Thus, EVs do not alter the fundamental mechanism of miRNA-mediated gene regulation but extend its spatial influence, converting localized radiation responses into coordinated multicellular adaptation [47,48,49,50,51]. Importantly, EV-mediated miRNA communication is a selective and dynamic process influenced by cargo sorting mechanisms and post-transcriptional modifications, which may determine whether transferred miRNAs exert protective or sensitizing effects in recipient cells [52].

Collectively, ceRNA networks regulate the intracellular availability and functional activity of miRNAs, whereas EVs determine their intercellular distribution and communication range. Together, these mechanisms establish a multilayer regulatory axis that links intracellular radiation adaptation with multicellular responses, thereby shaping tumor radiosensitivity and therapeutic outcomes.

**Table 2:** Representative non-coding RNA networks and extracellular vesicle-associated miRNAs involved in radiation responses.

Category	Molecule/Regulatory Axis	Biological Role in the Radiation Response	Experimental Model
lncRNA-mediated ceRNA regulation	NORAD/miR-199a-5p/EEPD1/ATR–CHK1 [35]	Enhances HR and promotes DNA damage repair.	Esophageal squamous cell carcinoma
	TRERNA1/miR-22-3p/SP1 [36]	Facilitates DSB repair and contributes to radioresistance.	Non-small cell lung cancer
	PVT1/miR-9-5p [38]	Promotes autophagy and suppresses apoptosis, thereby enhancing radioresistance.	Colorectal cancer
	HOTAIR/miR-93/ATG 12 [37]	Promotes autophagy and suppresses apoptosis, thereby enhancing radioresistance.	Colorectal cancer
	NEAT1/miR-448/GSDME [39]	Regulates radiation-induced pyroptosis.	Colorectal cancer
	FTX /miR-625-5p/SLC7A11 [40]	Suppresses ferroptosis and promotes radioresistance.	Radioresistant colorectal cancer cells
circRNA-mediated ceRNA regulation	circTP53, circFBXW7, and circATAD2 [41]	Promote autophagy and inhibit apoptosis, thereby supporting cell survival after irradiation.	Triple-negative breast cancer
	circ_0006174/miR-940/IGF1R [42]	Activates pro-survival signaling and contributes to radioresistance.	Colorectal cancer
	circ-TTC17/miR-145-5p/SIRT1 [43]	Suppresses autophagy and modulates cellular radiosensitivity.	Esophageal squamous cell carcinoma
	circ_101491/miR-125a-5p [44]	Promotes radiation-induced apoptosis and enhances radiosensitivity.	Esophageal squamous cell carcinoma
	circRAB12/circBACE2/circBIRC6/let-7/ITGB3 [45]	Promote migration, invasion, and bone metastasis following radiotherapy.	Triple-negative breast cancer
	circFOXO1/miR-27a-3p/BNIP3 [46]	Promotes autophagy and suppresses apoptosis, thereby enhancing radioresistance.	Triple-negative breast cancer
EV-associated miRNA regulation	EV-miR-21-5p/CDC25A [47]	Prolongs G1 arrest and facilitates DNA damage repair in recipient cells.	Vascular endothelial cells
	EV-miR-769-5p/TGFBR1 [48]	Amplifies the RIBE in non-irradiated recipient cells.	Ultraviolet- or radiation-induced bystander models
	EV-miR-132-3p, modification-dependent [52]	Exerts modification-dependent effects: the unmodified form promotes radiation injury, whereas the 3′-triuridylated form facilitates DNA repair.	Esophageal epithelial cells
	EV-miR-1246/LIG4 [14]	Inhibits NHEJ and propagates DNA damage to recipient cells.	Bronchial epithelial cells
	EV-miR-3160-5p/RGMB [49]	Suppresses the metastatic potential of recipient cancer cells.	Pancreatic cancer cells
	EV-miR-193a-3p/PTEN/Akt [50]	Promotes epithelial–mesenchymal transition, migration, invasion, and lung metastasis.	Esophageal squamous cell carcinoma
	EV-miR-34a-5p/CDKN1A [51]	Induces ferroptosis in non-irradiated recipient B cells.	Human B lymphocytes

## 4. Applications of miRNAs in Radiation Medicine

**Figure 3:**
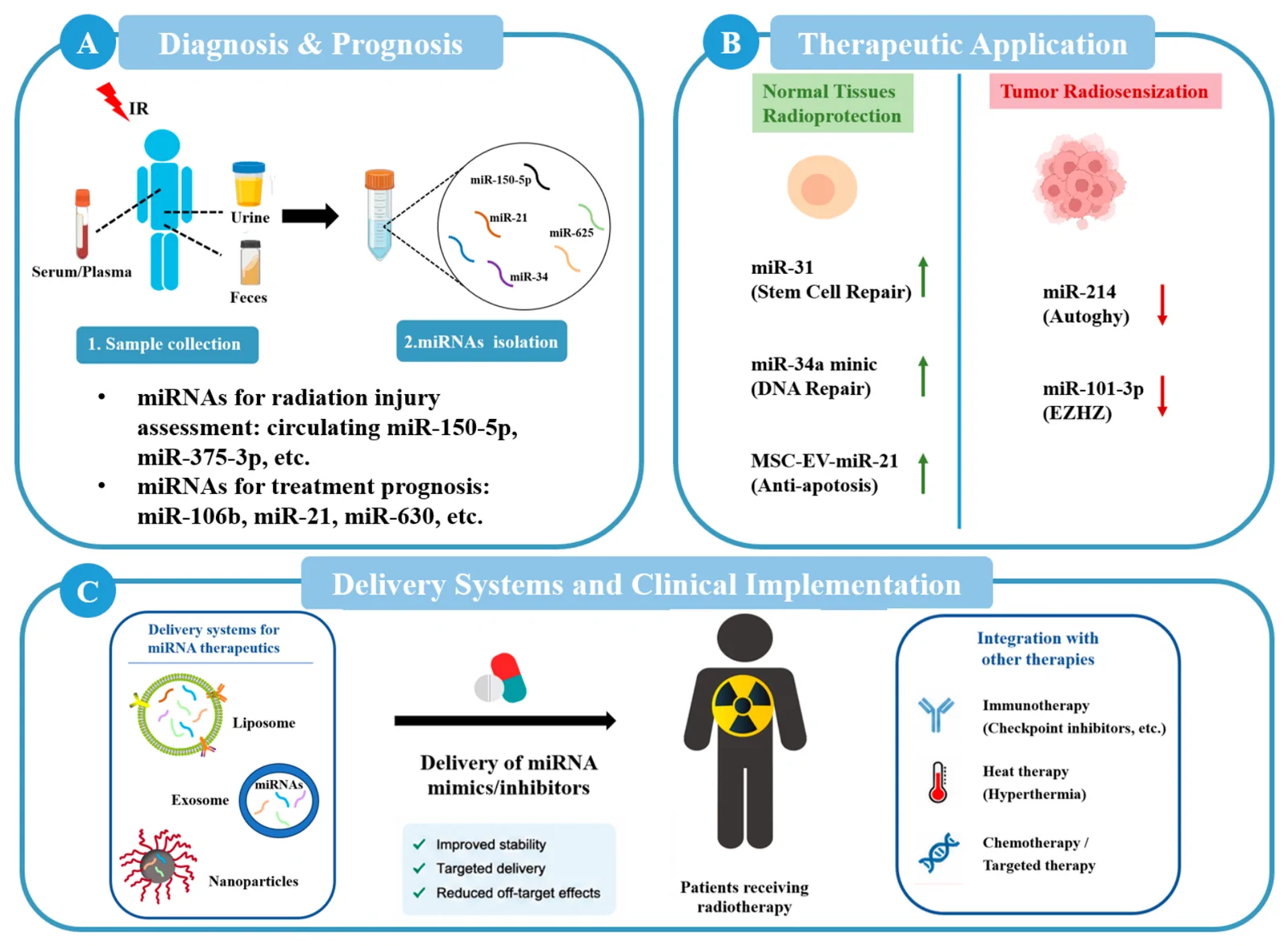
Clinical applications of miRNAs in radiotherapy. (**A**) miRNAs can be detected in biofluids (serum/plasma, urine, feces) from patients receiving radiotherapy. Their expression profiles are used for radiation injury assessment and treatment prognosis. (**B**) miRNAs can apply to protect normal tissues or sensitize tumors to radiotherapy. (**C**) Delivery systems enable efficient administration of miRNA therapeutics, which can be combined with other treatment modalities to achieve synergistic effects and improve clinical outcomes. Arrows indicate changes in miRNA expression levels, with ↑ representing upregulation and ↓ representing downregulation.

Given their central roles in regulating radiation responses, miRNAs are increasingly being translated from basic research into clinical applications; as biomarkers, they show great promise in the assessment of radiation damage, the prediction of radiotherapy efficacy, therapeutic interventions and precision delivery (Figure 3).

### 4.1. Radiation Injury Assessment

A critical priority following radiation exposure is the rapid estimation of absorbed dose and identification of affected organs. Ionizing radiation induces dynamic, dose- and time-dependent alterations in intracellular miRNA expression profiles, a subset of which is subsequently released into circulating biofluids, including blood, urine, and EVs. Owing to their remarkable stability, non-invasive accessibility, and suitability for longitudinal monitoring, circulating miRNAs have emerged as promising candidates for radiation biodosimetry. Radiation-responsive miRNAs often exhibit tissue-specific expression patterns, reflecting injury to radiosensitive organs such as the hematopoietic system, gastrointestinal tract, kidney, and oral mucosa [53,54,55,56,57]; changes in the expression of certain circulating miRNAs are also closely correlated with the radiation dose and the extent of tissue damage, and may serve as potential biomarkers for dose stratification and damage assessment. For example, plasma miRNA profiles such as miR-150-5p, miR-342-3p and miR-146a-5p can distinguish localized radiation damage, with miR-139-5p and miR-532-5p being correlated with damage scores [58]. miR-126a-5p and miR-133a-3p are associated with low-dose radiation exposure, whilst miR-709, miR-155-5p, miR-2137 and miR-99a-5p are associated with high-dose radiation responses [59]. Recent studies have further demonstrated that multi-miRNA signature models outperform single biomarkers in distinguishing exposure levels, estimating absorbed dose, and predicting radiotherapy-associated toxicities [60,61]. In parallel, advances in detection platforms, including high-throughput sequencing and sensitive quantitative assays, continue to improve the accuracy, sensitivity, and practicality of miRNA-based diagnostics. These developments underscore the growing potential of miRNA profiling as a rapid, minimally invasive tool for real-time monitoring and clinical management of radiation injury [62,63].

### 4.2. Prediction of Radiotherapy Response and Prognosis

Beyond their utility in radiation injury assessment, miRNAs have emerged as promising predictive biomarkers for radiotherapy response and long-term clinical outcomes. Prior to treatment, distinct miRNA expression signatures reflect the intrinsic radiosensitivity of tumor cells, thereby enabling stratification of patients into radiosensitive and radioresistant subgroups [64,65]. During radiotherapy, dynamic fluctuations in miRNA levels provide real-time insights into the biological responses of both tumor and normal tissues to irradiation and are closely associated with treatment response and disease progression [50,66]. In the post-treatment setting, numerous miRNAs have been correlated with key clinical endpoints, including local recurrence, distant metastasis, and overall survival, supporting their application in prognostic evaluation and long-term patient monitoring [67,68,69,70]. Importantly, multi-miRNA predictive signatures derived from multi-center studies have demonstrated superior performance compared with individual miRNAs, offering enhanced robustness and accuracy in predicting therapeutic response. These findings highlight the potential of miRNA-based models as molecular tools to guide precision radiotherapy and individualized treatment strategies.

### 4.3. miRNA-Mediated Therapeutic Intervention

Beyond their diagnostic and predictive value, miRNAs represent attractive targets for therapeutic intervention in radiotherapy. miRNA mimics and inhibitors can be employed to modulate critical biological processes, including DNA damage repair, cell cycle progression, apoptosis, and oxidative stress, thereby enhancing tumor radiosensitivity. Conversely, certain miRNAs exert protective effects in normal tissues by attenuating radiation-induced damage, thus improving treatment tolerance and reducing adverse effects. This dual functionality underscores the unique potential of miRNA-based strategies to simultaneously achieve tumor radiosensitization and normal tissue protection. Recent studies have further explored the integration of miRNA-based modulation with other therapeutic modalities, including hyperthermia, sonodynamic therapy, and small-molecule agents. These combinatorial approaches enhance radiotherapy efficacy through coordinated multi-target and multi-pathway regulation [71,72,73,74,75,76,77,78,79,80,81]. Collectively, these advances position miRNAs as both key regulators of radiation responses and promising therapeutic targets in translational radiation medicine.

### 4.4. miRNA Delivery Strategies

Efficient and targeted delivery remains a critical prerequisite for the clinical translation of miRNA-based therapies. Currently, delivery systems can be broadly categorized into natural carriers and synthetic nanoplatforms. Among them, EVs, particularly those derived from mesenchymal stem cells, exhibit low immunogenicity, intrinsic targeting capacity, and high biocompatibility, making them attractive vehicles for miRNA delivery. These properties enable efficient transfer of functional miRNAs and have demonstrated considerable potential in both tumor radiosensitization and protection of normal tissues [82,83,84]. In parallel, synthetic delivery systems—including lipid nanoparticles, polymer-based nanomaterials, and functionalized inorganic nanocarriers—offer enhanced stability, targeting specificity, and controllable release profiles. Through rational design, such platforms can be engineered to achieve stimuli-responsive delivery and synergistic interactions with radiotherapy, thereby improving therapeutic efficacy across diverse tumor models [85,86,87]. With continued advances in biomaterials and nanotechnology, miRNA delivery strategies are evolving toward greater precision, controllability, and clinical applicability, laying a solid foundation for future translational applications (Table 3).

Overall, miRNAs span the full continuum of radiation medicine, from injury assessment and response prediction to therapeutic intervention and targeted delivery. By integrating molecular regulation with clinical application, miRNAs represent a critical bridge linking fundamental radiation biology with precision radiotherapy.

## 5. Limitations and Future Directions

Although miRNAs show great promise for application in the field of radiation medicine, their translation from basic research to clinical practice still faces a range of challenges, including elucidating the underlying mechanisms and clinical implementation.

### 5.1. Limitations in Mechanistic Studies

miRNA regulatory networks are highly complex. A single miRNA can act on multiple target genes simultaneously, whilst a single target gene may also be co-regulated by multiple miRNAs, forming complex regulatory networks [92]. This multi-target regulatory pattern makes it difficult to accurately predict the specific role of miRNAs within the DDR network, and interventions targeting a single miRNA may result in unintended effects. Furthermore, miRNA function is markedly context-dependent. The same miRNA may exert different or even opposite effects in different tissues, cell types and physiological states, with its regulatory effects influenced by factors such as the target gene background, cellular environment and disease status [93,94]. At present, most studies are still based on specific cell models and single experimental conditions, and there remains a lack of systematic understanding of the role of miRNAs in complex tissue environments and during dynamic radiation response processes.

### 5.2. Barriers to Clinical Translation

Although numerous radiation-responsive miRNAs hold potential as biomarkers and therapeutic targets, their clinical application remains subject to a range of limitations. Firstly, miRNA expression levels are influenced by factors such as genetic background, age, sex and epigenetic modifications, as well as hypoxia, inflammation, viral infection and the immune status within the tumor microenvironment [20,85,95,96,97,98]; simultaneously, radiation dose, irradiation method and the type of irradiated tissue may also alter their expression patterns [3,54,58], leading to certain discrepancies between different studies. Secondly, there is a lack of uniform standards for the detection of circulating miRNAs. Different studies vary in terms of sample source, RNA extraction, detection platforms and data normalization methods; in particular, miRNAs derived from serum, plasma and exosomes may exhibit distinct expression profiles, thereby limiting the comparability of research findings and their clinical application [99,100]. Furthermore, the therapeutic application of miRNAs continues to face challenges regarding delivery efficiency and safety. As miRNAs are susceptible to degradation by endogenous nucleases and exhibit limited cellular uptake efficiency, achieving effective enrichment in target tissues remains a key challenge [101]. Although the nanocarriers and exosome delivery systems currently under development have enhanced the delivery potential of miRNAs, issues regarding targeting, immune responses and long-term safety still need to be addressed. The first miRNA drug, MRX34, had its clinical trials terminated due to severe immune-related adverse reactions, further illustrating the need to refine the safety evaluation system for miRNA therapy [102,103].

### 5.3. Future Directions

Future developments in the field of miRNA in radiation medicine need to gradually shift from single-molecule studies towards a systematic, dynamic and precision-based research model. Firstly, high-resolution miRNA-DDR regulatory network maps could be constructed by integrating single-cell sequencing, spatial transcriptomics and proteomics technologies. Utilizing artificial intelligence and machine learning algorithms, the interaction patterns between miRNAs and key nodes in the DDR could be predicted, and their functional heterogeneity across different cell types and stages of damage analyzed, thereby identifying more specific key regulatory nodes. Secondly, further exploration can be undertaken into how miRNAs act as bridges linking cellular metabolic states (such as mitochondrial function and oxidative stress) with DNA repair capacity. Elucidating how metabolic reprogramming influences DDR efficiency via miRNA mediation will provide new strategies for ‘metabolic-genetic’ dual intervention in cancer treatment. Thirdly, novel nanocarriers with high biocompatibility, tissue specificity and stimulus responsiveness should be developed to enhance the enrichment efficiency of miRNA mimetics or inhibitors in target tissues. Concurrently, by integrating the CRISPR/Cas system or base editing technology, precise regulation of endogenous miRNA genes can be achieved, thereby avoiding the uncontrollable risks associated with exogenous introduction and improving the safety and efficacy of treatment. Finally, a DDR functional assessment system based on miRNA expression profiles will be established to serve as a biomarker for predicting sensitivity to radiotherapy, resistance to chemotherapy and prognosis. Large-scale clinical trials will be conducted to validate the efficacy of miRNA modulators in combination with conventional radiotherapy and chemotherapy, and personalized treatment plans will be formulated based on patients’ miRNA profiles, thereby facilitating a transition from ‘one-size-fits-all’ to ‘precision’ treatment.

## Figures and Tables

**Table 1:** Representative radiation-responsive miRNAs involved in DDR and cell fate determination.

Category	miRNA	Target (s)	Biological Role
Cell cycle checkpoint regulation	miR-16-5p [9,27]	Cyclin D1/E1; PI3K/AKT/mTOR	Induces G1 arrest and suppresses survival signaling, thereby enhancing radiosensitivity.
	miR-21-5p [10]	CPEB3/CDK1/Cyclin B	Maintains G2/M arrest and contributes to radioresistance.
	miR-101-3p [11,28]	Wee1; BIRC5	Abrogates G2/M arrest, promotes apoptosis, and enhances radiosensitivity.
	miR-30a [29]	ATM	Weakens the G2/M checkpoint by suppressing ATM, leading to radiosensitization.
	miR-4443 [30]	PTPRJ	Regulates G2/M checkpoint progression and DNA repair efficiency, thereby promoting radioresistance.
DNA damage repair	miR-21-5p [16]	PARP1; POLQ	Promotes Alt-EJ, thereby contributing to radioresistance.
	miR-205 [31]	SK2; ZEB2/STAT3/MDM2	Suppresses DNA repair signaling and enhances radiosensitivity.
	miR-145 [13]	DNA-PKcs	Inhibits NHEJ-mediated DNA repair and enhances radiosensitivity.
	miR-4727-5p [15]	LIG4	Impairs DNA ligation during NHEJ, resulting in increased radiosensitivity.
	miR-200c-3p [32]	CBX5	Suppresses HR repair and enhances radiosensitivity.
	miR-320a [26]	RAD51; GPX4	Inhibits HR while promoting ferroptosis, thereby enhancing radiosensitivity.
	miR-4796 [8]	ATM; BRCA1; PARP1; RAD51	Simultaneously suppresses multiple DNA repair pathways, leading to radiosensitization.
	miR-384 [33]	ATM; Ku70/Ku80	Inhibits DNA damage repair and increases radiosensitivity.
Apoptosis regulation	miR-378a-5p [17]	LRP8/β-catenin axis	Promotes radiation-induced apoptosis and enhances radiosensitivity.
	miR-128-3p [18]	VEGFC; NF-κB pathway	Facilitates apoptosis and improves radiosensitivity.
	miR-7-5p [19]	XIAP; MCL1; REL; BIRC3	Activates apoptotic signaling by suppressing multiple anti-apoptotic proteins.
	miR-34a [20]	Bcl-2; MAPK pathway	Promotes apoptosis in a context-dependent manner.
	miR-222 [21]	BCL2L11	Suppresses apoptosis and contributes to radioresistance.
Autophagy and ferroptosis regulation	miR-214 [22]	ATG12	Suppresses autophagy and promotes radiosensitization.
	miR-183-5p [23]	ATG5	Maintains cytoprotective autophagy and reduces radiosensitivity.
	miR-22 [24]	REDD1	Suppresses autophagy while promoting apoptosis.
	miR-200c [25]	EGFR signaling	Promotes apoptosis and autophagic cell death, thereby enhancing radiosensitivity.
	miR-450a-5p [34]	DUSP10; p38/SAPK/JNK	Inhibits autophagy and promotes apoptosis, resulting in radiosensitization.

**Table 3:** Translational applications of miRNAs in radiation medicine.

Application	Representative miRNAs	Clinical Role	References
Radiation injury assessment	miR-150-5p, miR-375-3p, miR-1224-3p, miR-21, etc.	Enable assessment of radiation exposure, dose stratification, and organ-specific injury	[53,54,55,56,57,58,59]
Multi-miRNA signature models	miR-150-5p/miR-30b-5p/miR-320c; miR-148b-3p/miR-301a-3p/miR-423-3p	Improve accuracy in exposure identification, dose estimation, and prediction of radiotherapy-associated toxicity	[60,61]
Prediction of radiotherapy response	miR-20a-5p, miR-128-3p, miR-92a, miR-125b	Predict tumor radiosensitivity and radioresistance	[64,65]
Prognostic evaluation after radiotherapy	miR-208a, miR-630, miR-4776-5p, miR-193a-3p, miR-150-5p	Predict recurrence risk, overall survival, and treatment outcomes	[50,66,67,68,69,70]
miRNA delivery platforms	EVs; lipid nanoparticles; polymer-based nanocarriers	Enhance miRNA stability, targeting efficiency, and translational potential in radiotherapy	[82,83,84,85,86,87]
Normal tissue protection	miR-31, miR-34a, miR-21, miR-338-3p, etc.	Attenuate radiation-induced injury in normal tissues, including the gastrointestinal tract, hematopoietic system, and skin	[77,78,79,80,81,83,88,89,90,91]

## Data Availability

No new data were created or analyzed in this study. Data sharing is not applicable to this article.
